# Application of Chemometrics in Biosensing: A Brief Review

**DOI:** 10.3390/bios10080100

**Published:** 2020-08-17

**Authors:** Ekaterina Martynko, Dmitry Kirsanov

**Affiliations:** Applied Chemometrics Laboratory, Institute of Chemistry, St. Petersburg State University, St. Petersburg, 198504 Peterhoff, Russia; ekaterina.martynko@gmail.com

**Keywords:** biosensor, chemometrics, multivariate regression, classification, PCA, PLS, ANN

## Abstract

The field of biosensing is rapidly developing, and the number of novel sensor architectures and different sensing elements is growing fast. One of the most important features of all biosensors is their very high selectivity stemming from the use of bioreceptor recognition elements. The typical calibration of a biosensor requires simple univariate regression to relate a response value with an analyte concentration. Nevertheless, dealing with complex real-world sample matrices may sometimes lead to undesired interference effects from various components. This is where chemometric tools can do a good job in extracting relevant information, improving selectivity, circumventing a non-linearity in a response. This brief review aims to discuss the motivation for the application of chemometric tools in biosensing and provide some examples of such applications from the recent literature.

## 1. Introduction

**Figure 1:**
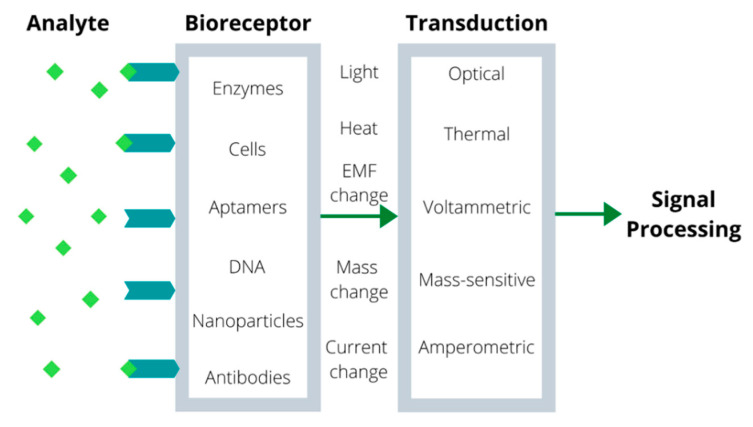
Schematic representation of a biosensor.

Biosensors combine bioreceptor recognition with various physicochemical transduction principles, such as amperometry, voltammetry, potentiometry, different optical techniques, etc. [1]. Figure 1 shows a schematic representation of a biosensor with the existing bioreceptor types and transduction techniques.

The most important feature of biosensors in comparison to chemical sensors is the possibility to achieve extreme selectivity with the appropriate bioreceptor for the analyte of interest. The wide variety of bioreceptors (such as antibodies, aptamers, molecularly imprinted polymers, DNA, etc.) and techniques for their immobilization allows the creation of highly sensitive biosensors for a broad range of analytes.

**Figure 2:**
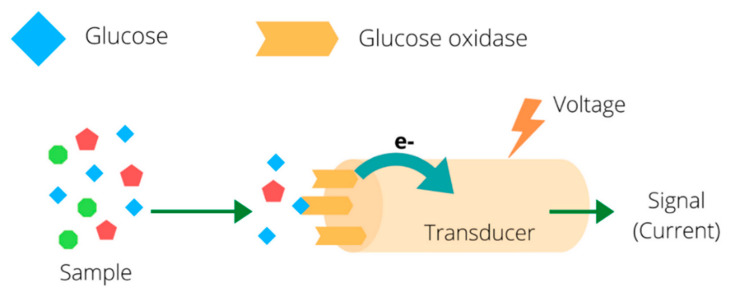
Scheme of an amperometric glucose biosensor.

One of the first and probably the most widely used example of an enzyme biosensor is a device for glucose detection [2]. Such devices are based on the enzymatic transformation of glucose into gluconic acid assisted by glucose oxidase (GOx) [3] and the subsequent monitoring of either the amount of consumed oxygen or obtained hydrogen peroxide. Various transduction principles may be applied for glucose sensing, but the most widespread are amperometric glucose biosensors (Figure 2).

The creation of the first enzyme-based glucose biosensor by Clark and Lyons in 1962 initiated the development of the entire field of biosensors. Since then, enzymes remain the most popular bioreceptor type for the detection of glucose and other analytes.

Due to the high selectivity of biorecognition elements, the calibration of a biosensor typically does not require using some sophisticated mathematical tools, and ordinary univariate regression can be employed to relate the response of the sensor with the analyte concentration. At the same time, when trying to resolve complex mixtures of several structurally similar analytes in real matrices, various interferences may occur, leading to the increase in analytical errors. The first possible option to circumvent this is to design more advanced and selective recognition elements and sensing platforms; however, this way typically requires significant investments of time and effort. Another option is to use more sophisticated data processing techniques that would still allow the construction of reliable calibration models, even with non-ideally selective sensor responses. As “math is cheaper than physics,” this option appears to be more attractive, and it builds a solid motivation for the application of advanced multivariate mathematical modeling tools in biosensing. This latter research field (also known as chemometrics) represents a rapidly developing part of modern chemical science. The application of chemometric tools in biosensing can provide several benefits: (1) the use of experimental design methodology can significantly reduce the cost of sensor composition optimization; (2) the application of multivariate data visualization tools, such as principal component analysis (PCA), can provide useful insights into the experimental data; (3) multivariate regression methods, including partial least squares regression (PLS) and artificial neural networks (ANN), allow for the effective handling of non-ideal analytical signals impacted by non-linearities, interferences, measurement noise.

Moreover, the use of chemometrics gave birth to the so-called “bioelectronic tongues”—arrays of biosensors [4] where several sensing elements with overlapping sensitivity patterns are employed simultaneously to increase the analytical performance of the system. Another relevant field in biosensor research where advanced mathematical modeling can be beneficial is quantitative structure–property relationships (QSPR)—the approach allowing for the prediction of sensor performance based on new sensing elements without physically producing these elements and based solely on the chemical structures of the active components. Unfortunately, this technique, which is well established in other research areas, such as drug discovery [5] and toxicity evaluation [6,7], has not been extensively explored for biosensing yet, and there are only a few examples in literature.

The purpose of this review is to give the basic ideas about the benefits that one may get using chemometrics in biosensor research and to promote a wider application of chemometric tools in biosensing. In no way, the authors aim to embrace the whole body of literature available on the topic, but they try to provide some convincing examples instead. The paper is organized as follows: first, we will introduce several typical chemometric tools explaining their prerequisites and outcomes using examples from literature; then we will review the common examples from different fields like environmental monitoring, food, and beverage analysis, biomedical applications; finally, the view of the authors on the perspectives and future trends in chemometric processing of biosensor data will be provided.

## 2. Typical Chemometric Tools Employed in Biosensor Research

**Figure 3:**
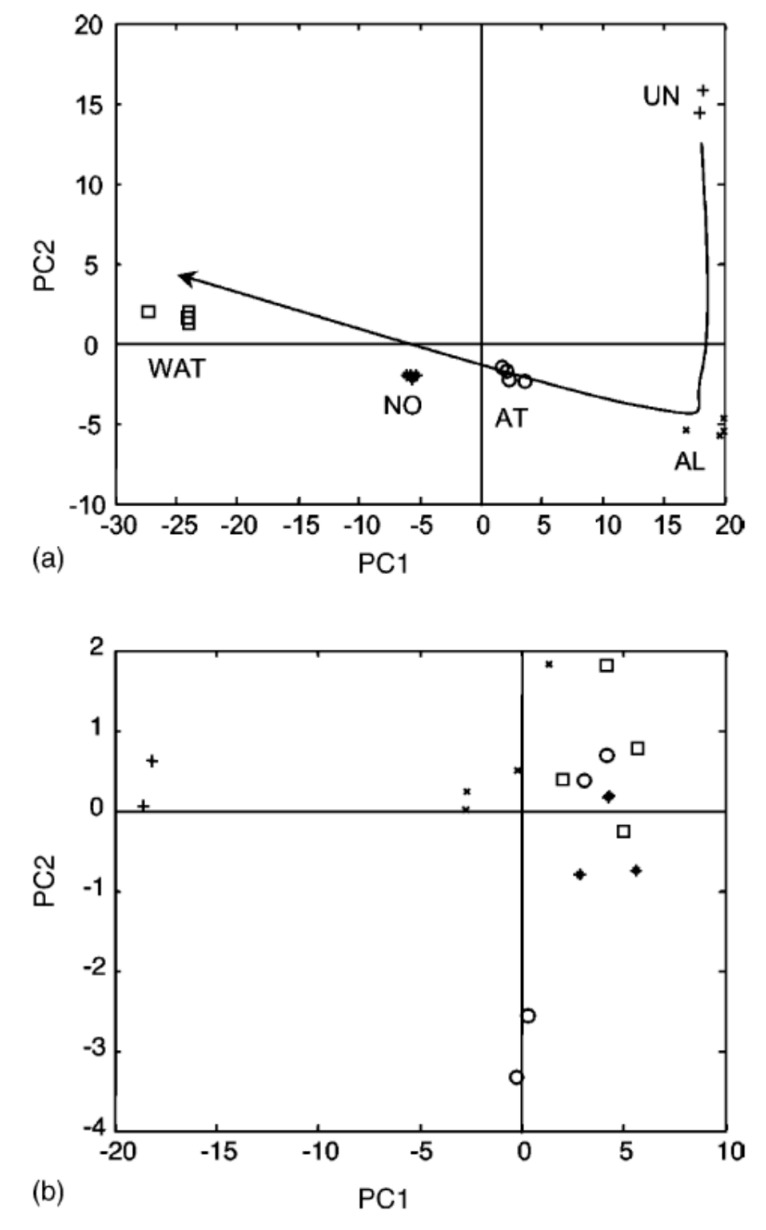
Principal component analysis (PCA) score plots obtained with (**a**) only two sensors from the array, (**b**) with the other six sensors. (+) Untreated; (×) alarm; (○) alert; (•) normal; (□) water. Reproduced from [9] with permission from Elsevier.

Let us now briefly introduce several of the most popular chemometric tools employed in biosensing. At first, it is crucial to understand the data structure required for chemometric processing. Unlike with conventional ***univariate*** calibration approaches where biosensor response in each sample is characterized by a single number (e.g., the current value, or the voltage value), chemometrics is dealing with ***multivariate*** data. In this case, the response of biosensor (or the set of several biosensors) is given by the set of numbers (e.g., voltage values registered at different currents or the responses of several sensors simultaneously). In the latter case, each measured sample can be represented as a point in a multidimensional space with dimensionality defined by the number of values in the registered response. The basic method of chemometrics is principal component analysis (PCA), which is primarily aimed at the convenient visualization of multivariate data and finding specific patterns therein [8]. PCA is based on projecting the initial data points from multivariate space into the space of smaller dimensionality, which is formed by the new coordinate axes—so-called principal components (PCs). The first PC is drawn in the direction of maximal variance in the data, the second PC covers the next direction of maximal variance orthogonal to the first PC, and so on. This procedure allows for constructing the PCA score plot where the initial samples from multivariate space are depicted in the two-dimensional space typically given by PC1-PC2. An interesting feature of this plot is that the similar studied samples will be shown as neighboring points in this plot, while dissimilar will be located at a certain distance (the more they differ, the longer the distance). As an excellent example, let us consider the work by Tønning et al. [9], where biosensor array based on eight platinum sensors treated with different enzymes was applied for evaluation of wastewater quality. The readings of each sensor were collected at 400 time channels yielding a truly multivariate response. The analyzed water samples were of five different types: untreated, alarm, alert, normal, and pure water. PCA processing of the multivariate response of biosensor array yielded the score plots shown in Figure 3. The authors found that not all of the sensors from the array contribute equally to the recognition of water quality and the PCA score plot constructed with the response of six sensors does not provide the separation of samples according to water type (Figure 3b), while the use of just two sensors yields distinct grouping according to water type (Figure 3a).

In this way, PCA can be applied to study the ability of individual sensors and multisensor arrays in distinguishing between different types of complex samples. While PCA is purely a visualization tool and suits solely for the exploratory data analysis, many chemometric tools allow numerical predictions of sample quality parameters. The most popular one is probably partial least squares regression (PLS) [10]. This is the method of multivariate regression aiming at relating the multivariate biosensor response to the analyte concentration values. Just like with ordinary least squares regression ***y* = *b*_0_ + *bx*** the method seeks the coefficients b for the equation ***y* = *b*_0_ + *b*_1_*x*_1_ + *b*_2_*x*_2_ + *…* + *b_i_x_i_*** that will convert the response values (***x*_1_*, x*_2_*,…, x_i_***) into the analyte concentration ***y***. Once these coefficients are found (the calibration is performed)—one can employ them for calculating ***y*** values in new samples. It must be noted that PLS, in general, allows for relating the sensor signal with any meaningful parameter ***y*** characterizing the sample (not necessarily a concentration), like, e.g., its toxicity, or intensity of particular taste descriptor in terms of the human sensory panel. There are different mathematical ways of calculating ***b*** coefficients, and the PLS algorithm finds them in the projection space similar to that of PCA with the only difference that PLS components are drawn in the direction of maximal variance in response space, which is correlated with the variance in calibration values of ***y***. This is a very powerful feature of PLS, allowing extracting useful chemical information from noisy, overlapped, or otherwise distorted analytical signals. The results of PLS modeling are often presented as a “measured vs. predicted” plot indicating reference values of the modeled parameter on the *x*-axis and the values predicted from the model on the *y*-axis. An ideal case of complete correspondence between the two, this will be a straight line originating from the center of the coordinate system and having the slope of 45°. The RMSEP—root-mean-square error of prediction—is normally applied to judge the predictive performance of the model:

$$RMSEP=\;\sqrt{\frac{\sum {\left(y_{i,ref}-y_{i,pred}\right)}^{2}}{n}},$$

where ***y_i,ref,_*** and ***y_i,pred_*** are ***y*** values for the ***i*th** sample obtained from the reference method and the model correspondingly, and ***n*** is the number of samples. The RMSEP has the units of the modeled parameter and should always be reported together with the range of the modeled parameter to assess practical applicability.

**Figure 4:**
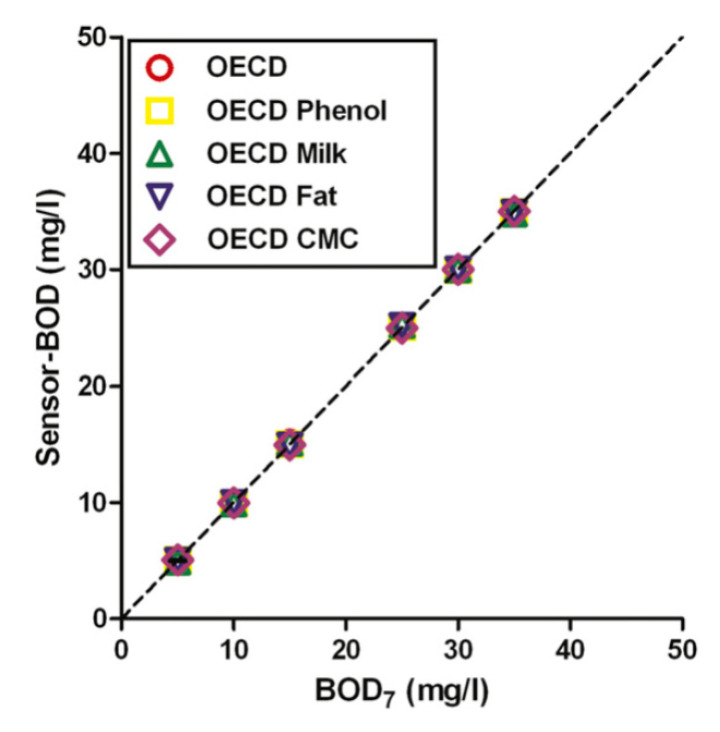
Measured vs. predicted plots for PLS models relating the response of biosensor array based on different semi-specific and universal microorganisms for the evaluation of biochemical oxygen demand (BOD) measurements in various synthetic industrial wastewaters. Reproduced from [11] with permission from Elsevier.

A typical example of PLS application in biosensing can be found in the study by Raud and Kikas [11], where biochemical oxygen demand (BOD) in simulated industrial wastewaters was assessed using the biosensor array. The idea behind this study was to substitute the time-consuming BOD evaluation procedure that takes seven days with much faster biosensor analysis. It is important to mention that BOD values are not related to the concentration of some particular individual chemical components, but they reflect the total content of the bio-oxidizable organic matter. As such, it is an important parameter to judge the overall water quality. It can be seen (Figure 4) that the developed biosensor array gives quite precise estimates of BOD values, and the PLS-predicted BOD differed from BOD_7_ by less than 5.6% in all types of samples.

**Figure 5:**
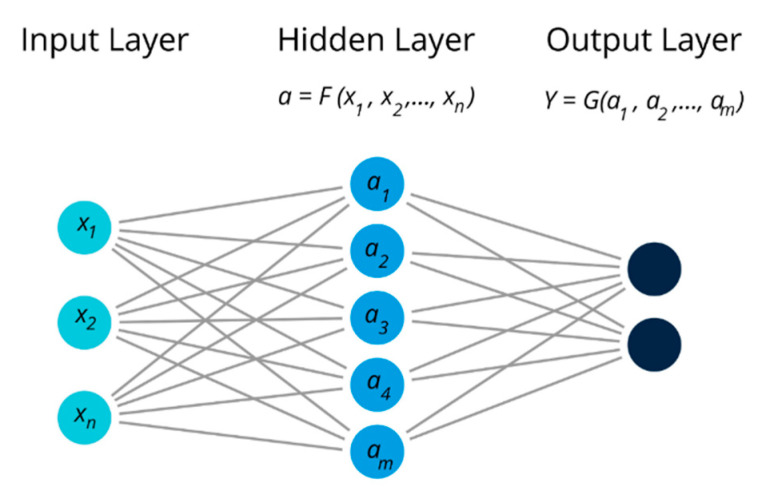
Schematic representation of the artificial neural network architecture.

Another powerful and popular data processing tool in chemometrics is the methodology of artificial neural networks (ANNs), the group of methods capable of handling both classification and numerical prediction tasks [12]. The basic ideas of ANN are coming from the simplified mathematical interpretation of education and recognition processes in the mammalians’ brain. The principal scheme of ANN is given in Figure 5. ANN consists of three types of layers: input, hidden, and output. There can be several hidden layers, and their particular architecture depends on the complexity of the addressed task. The simplest neural network has three layers—structural mathematical units that bear the numerical values of NN parameters (Figure 5).

The input layer neurons obtain as input the initial analytical data: spectral intensities, parameters of chromatographical peaks, responses of single chemical sensors (*x*_1_, *x*_2_, *…*, *x_n_*). The number of input neurons corresponds to the dimensionality of the initial dataset, e.g., if the initial spectrum was recorded as the absorbance at 100 different wavelengths, then the number of input neurons will be set to 100. The second layer of a neural network is called the hidden layer, where the elements *a*_1_, *a*_2_…*a_m_* are related to the input layer neurons via certain mathematical functions, *F*, which are called activation functions. These activation functions can be both linear and nonlinear, e.g., exponential, sigmoidal, sinusoidal, etc., and that makes ANN a highly adaptable approach. All the hidden layer neurons are connected to the output layer, where the number of neurons is equal to the number of modeled parameters *Y* in the initial dataset. So, if the ANN is created to predict the octane number in the petrol sample based on its near infra-red (NIR) spectrum, the output layer will have only one neuron with the predicted octane numbers. The goal of neural network training is to find an optimal number of hidden layer neurons *m* and the optimal activation functions *F* and *G* between layers to minimize the error of prediction for the parameter of interest.

Another chemometric tool that is popular in biosensing is linear discriminant analysis (LDA)—one of the first methods historically developed for the classification of multivariate data. It is widely employed for the construction of classification models to distinguish samples from different classes based on biosensor response. The algorithm is based on the search of hyperplane in the variable space, which will separate the classes. In case of only two variables, this hyperplane is a straight line. In case of three variables, this will be an ordinary three-dimensional plane. LDA is a probabilistic method estimating probability density functions in a multivariate way. These functions are similar to the well-known normal distribution and they can be described by the similar multivariate analogues of means and variances. Thus, LDA is based on the assumption about the normality of sample distribution in the classes and the assumption about similar variance in the classes. When the samples under analysis are plotted in the variable space, one can calculate the decision boundary based on the equiprobabilistic hyperellipsoids defined from Mahalanobis distances (Euclidean distances corrected for intercorrelation between the variables) of the samples from the class centroids—this will be given by the points of intersection of such ellipsoids belonging to the different classes. The sample in question will belong to the particular class depending on its location regarding the decision boundary.

It must be pointed out that many other chemometric tools can be applied for the processing of biosensing data; however, the three methods described above are the most common ones, and, in this review, we will limit the detailed discussion to PCA, PLS, and ANN only. The readers interested in a more comprehensive overview of the available chemometric tools and methods are referred to the specialized books [13]. This section is followed by the examples of the chemometric application in biosensing from several important fields: environmental monitoring, food and beverage analysis, biomedical applications.

It is also important to mention that most of the popular chemometric algorithms are absolutely universal tools for processing any type of multivariate data, regardless of their nature. Thus, different algorithms can be applied to the same problem and various types of biosensor responses can be treated with the same algorithm.

## 3. Application of Chemometrics in Different Fields of Biosensing

### 3.1. Environmental Monitoring

There are numerous examples of biosensors and sensor arrays proposed for environmental monitoring. In 2007, the array of enzymatic amperometric biosensors based on three types of acetylcholinesterase was introduced to measure the concentrations of dichlorvos and carbofuran in water. The chronoamperometric response of three AChE biosensors was measured in 22 samples of two pesticide solutions with random concentrations, and the percentage of inhibition was calculated for each of the three enzymes. Τhe training subset comprised 16 points, and the test subset had 6. For each enzyme, residual enzyme activities at nine different incubation times (1–15 min) in each pesticide mixture were recorded, resulting in 594 residual activity values as the input information for the ANN. The output layer was defined a priori: it had two neurons, one for each pesticide concentration (dichlorvos and carbofuran). The pesticide concentrations ranged from 0.029 to 1.56 nM for dichlorvos and 0.071 to 8.3 nM for carbofuran. ANNs were used to build a model and predict sensor responses to the simultaneous presence of the two pesticides in mixtures. The predicted values were in accordance with the spectroscopic reference data [14].

The same research group also presented the simple methodology for the simultaneous detection of several phenolic compounds (phenol, catechol, m-cresol) using the amperometric enzyme biosensor based on polyphenol oxidase [15]. The optimized ANN with a linear transfer function resulted in a good prediction ability for all three phenolic compounds (*r* > 0.988) on the external test set. Another approach to determine phenolic contaminants (hydroquinone and guaiacol) in water was recently proposed by Mendes and coworkers [16]. The tyrosinase-based enzymatic voltammetric biosensor was developed, and the PLS model with four latent variables was built to predict concentrations of two phenolic compounds simultaneously with *R*^2^ > 0.99 and low relative error (1.3 to 4.4%) using cyclic voltammograms with 220 variables as the input data.

### 3.2. Water Quality

Most of the biosensors employed for the water quality determination are based on the amperometric transduction principle. The range of analytes of interest includes pesticides, phenolic compounds, heavy metal ions, and other non-specific pollutants. Some papers also aim to quantify both the biochemical and chemical oxygen demand (BOD and COD, respectively) based on the combination of biosensors data and chemometric processing. It is important to mention that this type of quantification of integral quality parameters is only possible with sensor arrays and chemometric modeling as there is no way to construct a single sensor responding to the variety of chemicals responsible, e.g., COD. Table 1 represents relevant examples of biosensor applications and chemometric data processing techniques used in environmental water analysis. More examples and details can be found in specific reviews on water quality assessment, e.g., [17].

As can be seen from the examples above, chemometric processing can be applied both for handling the responses of individual biosensors and for responses of biosensor arrays. The most popular bioreceptors for ecological monitoring are enzymes as they allow a rather straightforward determination of harmful contaminants, such as phenols and pesticides.

### 3.3. Food and Beverages Analysis

Another area where a lot of examples of chemometric processing of biosensor data are available is food chemistry. Biosensors based on various transduction principles are employed to analyze food and beverages, and data processing and modeling methods include Principle Component Analysis, Artificial Neural Networks, MCR-ALS, Partial Least Squares, etc.

For a deeper understanding of biosensor applications in different areas of edible media analysis, more specific reviews are recommended [34,35,36,37]. There are also reviews specifically dedicated to the chemometric processing of food analysis data [38,39]. This review is intended to show specific examples of chemometric modeling in the safety determination and quality assessment of foods and beverages.

In the field of safety analysis, there exist numerous examples of biosensors developed for the determination of pesticides, herbicides, fungicides, heavy metals, and other toxic and harmful compounds in food to ensure its safety and compliance with standards and regulations. Similar approaches employing single biosensors and biosensor arrays are for the gustatory qualities’ analysis and detection of adulterations in food and beverages.

Table 2 summarizes the relevant examples of the biosensors and biosensor arrays used for the various analytical tasks in food chemistry with the employed chemometric methods and the brief descriptions of the sensor bioreceptor and transduction principle.

As can be seen from Table 2, several biosensors and biosensor arrays are suggested for the analytical tasks emerging in food chemistry. The samples of interest include wholefoods, soft drinks, and alcoholic beverages. Various methods of chemometrics, from simple linear regression to advanced artificial neural networks, are used to quantify both the contaminants and harmful substances, such as pesticides and antibiotics, and various compounds responsible for the taste of the product, e.g., phenolic compounds in wines or glucose and fruit acids in juices.

### 3.4. Biological and Medical Chemistry

Another field of use for biosensors is various biomedical studies: it is rather convenient to study different proteins in living organisms, tissues, and biofluids using the biorecognition principle that allows for high selectivity and specificity to the analyte. However, not only highly selective biosensors are developed; biosensor arrays with cross-selectivity are also widely employed to quantify single analytes, classes of analytes, and integral sample characteristics [53]. This technique is often referred to as differential sensing, which is very similar to the abovementioned electronic tongue/nose approach [54]. The main idea is to use an array of cross-reactive sensors and then process the obtained data using chemometrics methods, such as PCA, ANNs, LDA, or HCA.

A lot of biosensors developed for the biochemical and medical purposes are built for the recognition and quantification of various proteins: enzymes, kinases, cytokines, antibodies, etc. Recently, the approach to determine the activity of extracellular signal-regulated kinase (ERK) was suggested [55]. This type of kinases represents a particularly informative biomarker for the ERK-driven cancers, e.g., melanoma. A suggested facile and direct method to determine the ERK activity is based on the array of sulfonamide-oxine (SOX) peptides differential biosensors and further LDA to reduce the dimensionality of the data and SVM regression with a linear kernel to build a predictive model. The calibration model was built using the measured fluorescence of the SOX-peptides in 24 ERK-enriched samples of cell lysates in the concentration range of 0–6.4 nM of recombinant ERK1. The obtained model was able to quantify the ERK activity in tumor samples with the results comparable to those obtained by traditional western blot and immune-complex protein kinase assay protocols.

One more example of the fluorescent SOX-peptides biosensing array to quantify mitogen-activated protein kinases activity was introduced by Zamora-Olivares D. et al. [56]. The obtained data were processed with PCA and LDA to reveal some cross-reactivity among the peptides on the loadings plot. The predictive model was built using SVM regression and showed a satisfactory performance with the RMSEP of 6.6% on the independent test set in the concentration range 0–50 nM.

Miranda O. R. et al. proposed an enzyme-nanoparticle sensor array for the identification of proteins in human urine samples at the nanomolar level [57]. The sensor was constructed via the binding of cationic gold NPs (nanoparticles) to the β-galactosidase enzyme electrostatically, inhibiting enzyme activity. Analytes—proteins in the sample—release the enzyme, restoring its catalytic activity towards the fluorogenic substrate 4-methylumbelliferyl-β-d-galactopyranoside and amplifying the response. The fluorescent responses were analyzed with LDA and classified by the Mahalanobis distances of unknown samples to the centroid of the respective protein clusters in the canonical score plot, resulting in 92% accuracy of classification.

De M. et al. described a method for protein sensing in human serum samples with a biosensor based on the conjugates of nanoparticles with the green fluorescent protein (GFP) [58]. Fluorescent patterns of five serum proteins (human serum albumin, immunoglobulin G, transferrin, fibrinogen, and α-antitrypsin) were described, and, with the help of LDA, the proteins were identified with the accuracy of 100% in the buffer solution and 97% in human serum.

Another approach to protein classification in human urine was suggested by Motiei L. et al.: an array of cross-reactive optical biosensors was developed based on oligonucleotides, modified with fluorophores and protein-binding groups to identify different glutathione S-transferases [59]. LDA was used to search for the classification patterns in the obtained data, and the classification accuracy was 91% on the independent test set.

Another important type of analytes suitable for the biosensing in biochemistry are cells of different types: from bacteria to human tissues. A magnetoresistive biosensor was introduced for the BCG mycobacteria detection for the express diagnostics of tuberculosis [60]. The miniature sensor is a chip functionalized with immobilized capture antibodies, and magnetic nanoparticles functionalized with detection antibodies are added to the sample to bind the BCG bacteria selectively. When the magnetic NPs go near the biochip surface, a magnetoreceptive change is created by the magnetic stray fields, and a voltage drop is observed proportional to the bacteria concentration. The PLS regression model was calculated for the obtained voltages data after the signal correction procedures to achieve the limit of detection of 10.8 cells/mL, which is significantly lower than the traditional Ziehl–Neelsen sputum smear microscopy test.

An interesting approach to the cell type differentiation was suggested by Bajaj A. and coworkers [61]: gold nanoparticle-green fluorescent protein sensing arrays were created to identify mammalian cells on the basis of cell surface properties. The cells were added to sensing NPs-GFP solutions, and change in fluorescence was subjected to LDA. The classification based on Mahalanobis distances from the classes resulted in an accuracy of 96% in the independent test set.

One more option for cell identification based on the optical cross-reactive biosensor array was presented by Tomita S. et al. [62]. The sensing technique was based on the polyion complexes (PICs) between anionic enzymes and synthetic poly (ethylene glycol)-modified polyamines, recognizing “secretomic signatures” in cell cultures. The response patterns analyzed with LDA allowed for the lineage identification of the osteogenic and adipogenic differentiation of human mesenchymal stem cells with an accuracy of 94%.

Apart from proteins and whole cells as the substances for biosensing and chemometric processing, other analytes include lipids, nucleotides, neurotransmitters, and pharmaceutical substances. For example, an enzymatic biosensor based on a set of three deoxynucleoside kinases was proposed to monitor the nucleoside analogs in human plasma [63]. A Bayesian model based on the Michaelis–Menten kinetics equation was built to identify the substrates of interest in mixtures, providing a simple and fast detection method for nucleoside and nucleoside analog in physiological samples.

Another interesting approach was suggested for the quantification of glycerides and their regio- and stereoselective identification [64]. The sensing method was based on human and bovine serum albumin mixed with fluorescent indicators and olefin cross-metathesis reaction as a pretreatment to distinguish unsaturated compounds. The results from the array were processed with LDA to classify the glyceride species according to their structural features successfully.

A multielectrode array was suggested for the voltammetric quantification of neurotransmitters dopamine and norepinephrine in biological fluids in situ, i.e., without any pretreatment steps [65]. The electrodes were modified with chitosan and carbon nanotubes, and the data were used to build a PLS regression model with RMSEP < 3 µM for both of the studied neurotransmitters in the concentration range 1–25 µM.

## 4. Experimental Design and Mathematical Modeling

Chemometric methods not only are useful in terms of processing the existing data but also for planning the experiments and rationally designing new sensors. One example of the experimental design is the attempt to evaluate how much the experimental design influences the prediction ability of a chemometric model [66]. The noninvasive approach to glucose biosensing via NIR transcutaneous spectroscopy was studied and compared with the traditional invasive portable glucose meter as a reference method. The key factors that were changed in different experimental setups were the time delay between measurements, the body temperature of the test subjects, and methods of model validation. It was found that temperature change introduced the most significant errors.

Another paper [52] described the statistical mixture design in the creation of the voltammetric biosensor for the detection of chlorogenic acid in various coffee beverages. The sensor was constructed based on graphite oxide, platinum nanoparticles, and biomaterials obtained from the ascomycetous fungus, *Botryosphaeria rhodina*, namely laccase and botryosphaeran. A factorial design was used to optimize the operational parameters of the sensor and the most suitable pH for the measurements. The optimized biosensor is able to quantify the chlorogenic acid in coffee samples resulting in statistically similar values to those obtained by HPLC at the 95% confidence level.

The chemometrics-assisted approach to the design of the bioreceptors for the new sensors is widely used in the development of new molecularly imprinted polymers (MIPs) [67]. MIPs are quite popular sensitive elements in analytical chemistry because of their ability to bind the analyte of interest selectively. The important advantage of the MIPs is the high tunability of their parameters since MIPs are human-made substances that are synthesized by copolymerization of monomers and cross-linkers in the presence of template molecules. These templates are later removed, and the remaining recognition cavities in the polymer matrix are complementary in shape and size to the analytes of interest. The main drawback associated with the impressive selectivity in the case of successful MIP synthesis is that the optimization of such synthesis is time consuming and is based on the trial-and-error approach [68].

The chemometric-based approach to MIP design and synthesis seems to be an excellent opportunity to overcome this disadvantage; therefore, it has already been demonstrated on a number of different templates. It is based on statistical analysis and allows using the experimental data for MIP design. For example, Muzyka et al. [69] demonstrated the synthesis of rationally designed vancomycin-selective MIP nanoparticles, taking into the design such factors as the number of functional monomers in the polymerization mixture, irradiation time, polymerization temperature, and elution temperature. The composite face-centered design (CCF) was used to understand that the irradiation time is the most important, and the temperature is the least important parameter for the total yield. In addition, the response surface methodology was used to reduce the amount of the necessary experimental effort, resulting in the yield of MIP nanoparticles of 3.4 a.u. (25 mg) in the optimized conditions, which is the highest yield achieved so far in one synthetic cycle.

Other examples of rational MIP synthesis design include the two-factor experimental design for the (–)-ephedrine imprinted polymer [70], optimizing the polymerization time, reaction temperature, and the concentration of initiator, and composite face-centered design with a response surface methodology to optimize the synthesis of propranolol imprinted MIP combinatorial library [71]. There are also optimization strategies for MIP synthesis based on molecular modeling and computational chemistry (e.g., [72,73,74]), but they are beyond the scope of this review.

Only a few examples of the structure–property/activity relationships modeling in biosensors development exist. Thus, there was an attempt to describe the antigen–antibody interaction kinetics in the sensor chips [75], or the study focused on the development of a QSPR model using an associative ANN to predict the excitation wavelengths of boronic acid-based fluorescent biosensors [76]. Such studies are not only limited in number but also tend to rely on tiny datasets for the model formation, therefore leading to the narrow applicability domain of the obtained QSAR/QSPR model. Nevertheless, there are examples of successful QSAR/QSPR modeling in the closely related field of chemical sensors [77,78]. This approach is entirely new to the whole domain of chemical, biochemical and biological sensors. Still, the first obtained results on structure–property relationship modeling in search of new sensors with desired properties look promising and show a possibility for the development of new sensors tailored for a specific analytical task.

## 5. Future Perspectives

We believe that further research in the application of chemometric methods to biosensor data will be focused on several main topics. The employment of chemometric algorithms may help in the mass-production of biosensors to improve their interbatch reproducibility. This can be achieved by using the methods of multivariate calibration transfer between two non-identical sets of sensors. These non-identical sensors will produce different responses’ values in the same sample, thus invalidating the calibration and implying the necessity of frequent measurements in calibration solutions. Since standardization at the physical level is not always possible due to the natural variability in raw materials, the application of mathematical methods appears to be an attractive option in this respect. This also relates to the long-term stability of biosensor response in applications requiring continuous measurements—the chemometric algorithms are available to eliminate long-term response drifts of sensors and sensor arrays [79,80].

Another important issue with chemometrics relates to the fact that at the moment, only a few reports are addressing the development of formal analytical figures of merit like sensitivity, selectivity, detection limits for sensors and sensor arrays employing multivariate calibration [81,82]. This lack of formal description hinders both the comparison of different results between each other and an appropriate metrological standardization of the corresponding biosensors and biosensor arrays, thus delaying a massive acceptance and introduction of such methods into routine laboratory practices.

With the active development of various machine learning tools intended both for solving classification and regression problems, one can also predict their adoption in the field of biosensing; however, thorough precautions must be taken against a careless implementation of all newly developed mathematical tools just because they are new. Quite often, the choice of the optimal method is very task specific, and the understanding of all pros and cons is needed for the responsible application of mathematical tools in biosensing.

## 6. Conclusions

Although it may seem unnecessary at first glance due to the high selectivity of bioreceptors, the application of chemometric methods can provide significant improvements for the analytical performance of biosensors. In particular, the following advantages can be achieved: better precision in the resolution of complex analyte mixtures, better tolerance of sensor response towards interferences, and a possibility of quantifying complex integral sample quality parameters depending on many various (bio) chemical components. As part of the discussed applications of chemometric tools to biosensor data in environmental monitoring, food, and beverage analysis, biomedical problems reveal the high potential of this approach for further development of biosensing. The discussed applications of chemometric tools to biosensor data in environmental monitoring, food, and beverage analysis, biomedical problems reveal the high potential of this approach for further development of biosensing. We believe that future research in this area will bring a whole variety of new exciting opportunities for using biosensors.

## Figures and Tables

**Table 1:** Relevant examples of biosensors and bioelectronic tongues for the water analysis.

Analytes	Transduction Principle	Data Analysis	Bioreceptor Type	Description	# of Sensors (Channels)	Reference
**Pesticides detection**						
Paraoxon and carbofuran	Amperometry	ANNs	Enzyme	AChE inhibition	4	[18]
Dichlorvos and carbofuran					3	[14]
Dichlorvos and methylparaoxon				AChE inhibition, FIA system	3	[19]
Chlorpyriphos oxon, chlorfenvinphos and azinphos-methyl oxon				AChE inhibition	2	[20]
Carbaryl, phoxim	Spectrophotometry			AChE inhibition, subsequent reaction of thiocholine with 5,5-dithiobis(2-nitrobenzoic) acid	1	[21]
Dichlorvos, malaoxon, chlorpyrifos-oxon, chlorpyrifos-methyl-oxon, chlorfenvinphos, pirimiphos-methyl-oxon	Chrono-amperometry			AChE inhibition in an automated system	6	[22]
Classification of pesticides residues into three groups (carbamates, pyrethroids, organophosphates)	Potentiometry		Cells	Cellular sensors based on bioelectric recognition assay	1	[23]
**Phenolic compounds**						
Catechol and 4-chlorophenol	Amperometry	PLS	Enzyme	A tyrosinase-based sensor in a FIA system	1	[24]
Phenol, catechol, m-cresol	Linear sweep voltammetry	ANNs		Polyphenol oxidase-based sensor in a SIA system	1	[15]
Binary mixtures: phenol/chlorophenol, cathecol/phenol, cresol/chlorocresol, phenol/cresol		PLS		Tyrosinase- and laccase-based sensors	2	[25]
Catechol, m-cresol, guaiacol (in artificial wastewater)	Cyclic voltammetry	FFT *, ANNs		Sensors based on Tyr, Lac, and Cu NPs	4	[26]
**Metal ions**						
Cu^2+^, Cd^2+^, Pb^2+^	Square wave voltammetry	nPLS **	Peptide	Single Au electrode modified with three different peptides	1	[27]
Cd^2+^, Pb^2+^, Zn^2+^	Differential pulse adsorptive stripping voltammetry	FFT, ANNs	Peptide	Array of peptide-modified electrodes	3	[28]
Fe^2+^	Spectrophotometry	PARAFAC ^x^	Nanosheet MoS_2_	Fe^2+^/MoS_2_ oxidation catalysis to form highly fluorescent compound (DAPN)	1	[29]
K^+^, Tl^+^	Spectrophotometry	PLS	DNA and NPs	ssDNA-AuNPs catalysis of the oxidation of TMB with H_2_O_2_ to generate fluorescent compounds	8	[30]
**Water quality metrics**						
General wastewater quality	Amperometry	PCA	Enzyme	An array of enzymes immobilized onto C and Pt working electrodes	8	[9]
Organic pollutant indexes in wastewater		PCA, PLS	Enzyme	Immobilized enzymes onto working electrodes	16	[31]
Biochemical oxygen demand (BOD) in wastewater		PLS	Microorganism	Microorganisms immobilized on the surface of a Clark-type electrode	7	[11]
Chemical oxygen demand (COD)	Generated current	ANNs	Microorganism	Microbial fuel cells-based sensors	1	[32]

* FFT = Fast Fourier Transform; ** nPLS = N-way Partial Least Squares [33]; ^x^ PARAFAC = Parallel Factor Analysis.

**Table 2:** Examples of biosensors and biosensor ETs for the food and beverages analysis.

Analytes	Sample	Transduction Principle	Data Analysis	Bioreceptor Type	Description	# of Sensors (Channels)	Reference
Pesticides: chlorpyriphos-oxon and malaoxon	Milk	Amperometry	ANNs	Enzyme	AChE inhibition in an FIA system	2	[40]
Insecticides: captan	Apples	Cyclic voltammetry	PCA, regression analysis		AChE inhibition	1	[41]
Antibiotics: tetracycline and cefixime	Milk	Square wave voltammetry	PCA, ANNs	Amino acid monolayer	Screen-printed Au electrode modified with Au NPs and a self-assembled monolayer of cysteine	1	[42]
**Phenolic compounds**							
Catechol, caffeic acid, catechin	Wine	Cyclic voltammetry	ANNs	Enzyme	Tyrosinase and laccase biosensors combined with Cu NPs	4	[43]
Ferulic, gallic, sinapic acids	Beer		ANNs, PCA				[44]
Total phenolic content	Wine		ANNs, DWT * + PLS		Tyrosinase and laccase sensors with electron mediators	12	[45]
Total phenolic content and discrimination of grape varieties			PCA		Tyrosinase and GOx biosensors with electron mediators	6	[46]
Bacteria: *Salmonella typhimurium*	Pork	Electrochemical impedance spectroscopy	PLS	Antibody	*Salmonella* antibodies immobilized on the surface of a gold microelectrode	1	[47]
Glycoalkaloids: α-solanine and α-chaconine	Potato	Amperometry	ANNs	Enzyme	AChE inhibition	2	[48]
Glucose and ascorbic acid	Fruit juice	Linear sweep voltammetry	ANNs	Enzyme	GOx biosensors with metal catalysts in an SIA system	3	[49]
Melamine and urea	Milk	Amperometry	LR **		AChE inhibition on the Pt/ZnO/Chitosan bioelectrode	1	[50]
Chloropropanols	Soy sauce	Spectrophotometry	LDA, PLS	Protein	Differential optical sensing with serum albumins coupled with a fluorophore	3	[51]
Chlorogenic acid	Coffee	Square wave voltammetry	PCA	Fungus	The measurement of laccase production by funghi in different conditions	1	[52]

* DWT = Discrete Wavelet Transform; ** LR = Linear Regression.

## Abbreviations

AChE	Acetylcholinesterase
ANN	Artificial Neural Network
FIA	Flow Injection Analysis
ET	Electronic Tongue
GFP	Green Fluorescent Protein
GOx	Glucose Oxidase
HSA	Human Serum Albumin
HCA	Hierarchical Cluster Analysis
LDA	Linear Discriminant Analysis
MAB	Monoclonal Antibody
MCR-ALS	Multivariate Curve Resolution Alternating Least Squares
NPs	Nanoparticles
PCA	Principle Component Analysis
PLS	Partial Least Squares
RMSEP	Root-Mean-Square Error of Prediction
SIA	Sequential Injection Analysis
SVM	Supported Vector Machines
QSAR	Quantitative Structure-Activity Relationship
QSPR	Quantitative Structure-Property Relationship
